# “I Can’t Blame Mum”: A Qualitative Exploration of Relational Dynamics in Women With Female Genital Mutilation (FGM) in the United Kingdom

**DOI:** 10.1177/1077801221994913

**Published:** 2021-03-08

**Authors:** Rebecca J. Newton, Jennifer Glover

**Affiliations:** 1 University of Oxford, UK; 2 Coventry University, UK

**Keywords:** female genital mutilation, FGM, relational dynamics, qualitative, interpretative phenomenological analysis

## Abstract

Female genital mutilation (FGM) is conceptualized as an interpersonal act, commonly initiated by mothers. This study investigates relational dynamics among adult women who experienced FGM in childhood and have since migrated to the United Kingdom. A qualitative research design was employed, using semi-structured interviews and interpretative phenomenological analysis (IPA) with nine women. Three superordinate themes emerged: (a) “The ‘who to blame?’ conflict: Preserving goodness in parents”; (b) “Better or worse? Positioning the self in relation to others”; and (c) “Regaining power: Righting the wrongs.” Implications for understanding the relational consequences of FGM and the discontinuation of its intergenerational transmission are considered.

## Introduction

### Female Genital Mutilation (FGM)

FGM touches the lives of an estimated 200 million women and girls worldwide (United Nations Children’s Fund, 2016). The practice is defined as “all procedures that involve partial or total removal of the external female genitalia, or other injury to the female genital organs for non-medical reasons” (World Health Organization [WHO], 2018). There is substantial anatomical variation in its form, namely, “Type I”: the partial or total excision of the clitoris or clitoral prepuce; “Type II”: additional cutting of the labia minora and/or majora; and “Type III”: the cutting and stitching of the labia, almost totally sealing the vaginal opening. This third type is also known as “infibulation” or “pharaonic” circumcision and requires a “deinfibulation” or opening procedure prior to childbirth. “Type IV” is used to describe all other medically unnecessary alterations or scarring of the genitalia (see WHO, 2018, for medical typology).

The sociocultural context surrounding the practice varies considerably across regions, including the age at which it is performed, from newborns to teenagers. It may be performed covertly or celebrated as part of a wider initiation ceremony. Cutting the genitalia may represent a “rite of passage” into womanhood (Esho, 2012), believed to confer cleanliness, sexual morality, and conservation of virginity and marriageability (Bogale et al., 2014). Most commonly practiced in Africa, the Middle East, and Asia, FGM occurs globally within migrating communities, passed down through generations of women due to its cultural longevity and significance, despite the growth of fierce opposition and illegalization in more than 60 countries worldwide (Nabaneh & Muula, 2019).

The ancient tradition of “female circumcision” was transformed linguistically into “Female Genital Mutilation” (FGM) by Hayes (1975) to draw attention to its violence and to separate the construct from male circumcision. FGM is widely criticized for contributing to a pervasive patriarchal oppression of female sexuality and is accused of breaching human rights to bodily integrity, the rights of the child, the right to be free from gender discrimination, and the right to be free from torture and degrading treatment (Khosla et al., 2017). Guidelines published by the WHO outline the immediate dangers of FGM and long-term risks to sexual, obstetric, and psychological health. The guidance unequivocally recommends the discontinuation of the practice and for increased understanding of and support for those experiencing short- and long-term health consequences (WHO, 2016).

Notwithstanding its international recognition and role in the safeguarding of women and girls at risk, “FGM” discourse also has the potential to perpetuate stigmatization, disempowerment, alienation, and persecution of women (Njambi, 2004), particularly of those who migrate to nonpracticing countries. It is therefore important to seek a comprehensive understanding of women’s experiences and the consequences they face, as they see them, both directly from those living with FGM and indirectly with the additional impact of migration.

### Relational Consequences of FGM

FGM is an interpersonal act that occurs within families and perpetuates through intergenerational transmission, most commonly organized by mothers for their daughters (Farina & Ortensi, 2014). There is evidence that women who have experienced FGM are at higher risk of long-lasting traumatic symptoms and associated mental health problems throughout their lives (Mulongo et al., 2014), with an increased vulnerability for those with more extensive FGM, that is, Type III (Köbach et al., 2018). High rates of somatic symptoms (Ahmed et al., 2017) and sexual dysfunction (Berg & Denison, 2012; Rouzi et al., 2017) have also been reported, as well as a felt sense of having “something missing” (Kahn, 2016) or taken without their consent (Al-Krenawi & Wiesel-Lev, 1999).

Some women report a sense of betrayal by their families and communities (Isman et al., 2013). So-called “betrayal traumas” have been found to be more likely than life-threatening traumas to lead to harmful interpersonal consequences in adulthood due to a fundamental breakdown in trust in others and sense of psychic safety (Birrell et al., 2017). For some, FGM may represent a single-event trauma (Behrendt & Moritz, 2005). For others, it may be better conceptualized as a repeated trauma as the ensuing complications can be recurrent and re-traumatization can occur at significant life stages, such as virginity loss and childbirth (Battle et al., 2017), and during corrective surgeries such as deinfibulation and clitoral reconstruction (Abdulcadir et al., 2017). Reactions to repeated trauma can manifest widely in domains of attachment relationships, affect regulation, dissociation, cognitive distortion, behavioral regulation, and self-concept (Cook et al., 2017).

Furthermore, the well-documented health consequences of FGM are inextricable from the relational lives of women. Studies of sexual dysfunction show that couples report low sexual satisfaction, frequent pain and discomfort, and some women view sex as a duty to the husband rather than a pleasurable or desirable experience (Fahmy et al., 2010). The subsequent relational impact on the couple and/or marital life is not yet well understood. Connor et al. (2019) depicted a vicious cycle of chronic pain related to FGM in which experiences of sexual pain led to anxiety, tension, and avoidance of intimacy, thereby damaging the quality of the romantic relationship and increasing the likelihood of further pain in the next sexual encounter. Living with chronic pain is known to contribute to interpersonal strain, and family members too may feel powerless, alienated, distressed, and isolated (West et al., 2012).

Few studies to date have investigated women’s experiences of FGM in a relational framework. In a mixed-methods study, Vloeberghs et al. (2012) identified three subgroups of women coping differently with the effects of FGM: the “adaptives,” the “disempowered,” and the “traumatized.” “Adaptives” were troubled with physical and sexual complications but coped well by asking for help from others and engaging with religion. The “disempowered” felt angry, deflated, and helpless. They avoided activity and sex, and marital relationships suffered. The “traumatized” suffered the most severe pain and mental health disturbance. They avoided sex, became isolated, and held great anger toward their mothers. This raises the question of how the severity of impact and how individuals cope affect significant relationships such as with mothers and husbands.

Predictably, the mother–daughter relationship has been identified as a meaningful area of study regarding FGM. Schultz and Lien (2014) found that the presence of the mothers at the “cutting” could damage the mother–daughter relationship in the short term, but this later returned to normal as mothers acted to reduce traumatic reactions in their daughters by providing security, connection, and a role model for managing pain. The authors posit that this ruptured and repaired relational dynamic is culturally situated, and that women who have migrated outside of their original contexts are in a particularly vulnerable position.

Migrant women were interviewed in a grounded theory study that produced a model of psychosocial consequences of FGM (Glover et al., 2017). Again, the mother–daughter relationship was a common source of conflicted emotions for women, comprising not only anger and hatred, but also compassion and forgiveness. A widespread lack of trust in friendships and fear of rejection by men were also reported. In addition, some women reported difficulties bonding with or caregiving for their new babies following serious complications in childbirth, an area that has not previously been studied in FGM populations despite evidence of a higher risk of obstetric complications (Anikwe et al., 2019; Berg & Underland, 2013).

Finally, a thematic analysis of interviews with migrant women by Parikh et al. (2018) found that women considered themselves to be different on migration to the United Kingdom and wanted to align themselves with those they considered “normal.” They feared rejection by men and felt insecure and inadequate in romantic relationships. Once again, the mother–daughter relationship was conflicted: Women described feelings of distrust, blame, and betrayal, and, in some cases, a breaking down of this relationship as a result. Some women shifted blame onto “culture,” and mothers who expressed regret could be forgiven. The study demonstrates how qualitative research can elucidate conflicted family dynamics that cannot be captured through quantitative methods and recommends future research to investigate the intersectionality of FGM, minority status, and family relationships.

### Current Research Aim

Despite the interpersonal nature of the phenomenon, there remains a paucity of research into relational consequences in the FGM literature. This study aimed to address this gap by employing a qualitative methodology to explore the subjective intricacies of relational dynamics in a small number of adult migrant women in the United Kingdom who experienced FGM in childhood. It aimed to achieve a greater understanding of women’s relationships with their mothers, partners, and other important figures in their lives, in relation to their FGM.

## Method

### Design

The study used a qualitative research design, utilizing interpretative phenomenological analysis (IPA) and semi-structured interview methodology. IPA draws upon three philosophical stances of inquiry: phenomenology or the study of experience; hermeneutics, that is, the theory of interpretation; and idiography, which is the focus on the particular (Smith et al., 2009). This allows for close examination into how individuals make sense of significant life experiences through facilitating expression in its own terms, rather than in predefined categories, with the aim of understanding something new.

### Participant Inclusion Criteria

- Adult women (18 years or older);
- Experience of FGM in childhood (any type, self-identified);
- Able to understand the study remit and give informed consent.

### Recruitment Sites

Participants were recruited from two sites. The first was a specialist FGM clinic situated within a U.K. National Health Service (NHS) gynecology department in London, offering diagnostic services, deinfibulation procedures, and specialist care for pregnant and nonpregnant women with FGM. The second was a charity organization in Reading that supports women with FGM through group meetings, education, and awareness events and campaigns.

### Procedure

#### Recruitment

Convenience sampling was used to recruit women who met the inclusion criteria at the two sites. The study was advertised through verbal invitation by the service leads on an opt-in basis. A total of nine women participated in the study; five from the NHS clinic and four from the charity group. Some women declined on the basis of being unwilling to be audio recorded or not feeling ready to talk about their experience; however, it was not possible to systematically record data on refusals due to the recruitment method used and practical constraints of the settings. The sample size was considered optimal for the IPA methodology: large enough to identify themes and illustrate variation, and small enough to preserve the richness and depth of idiosyncratic lived experiences (Smith et al., 2009).

#### Demographic questionnaire

A brief demographic questionnaire was administered to gather basic information about participants at the start of each interview to situate the sample in their personal and familial contexts.

#### Semi-structured interview

A semi-structured interview schedule was developed in line with IPA principles in being open-ended, expansive, and aimed at capturing subjective experience while using prompts flexibly to steer the topic toward the research aims. Participants were asked about their experience of FGM and were invited to talk about how they thought about it in relation to important people in their lives. Interviews were conducted face-to-face by the primary researcher (a White British female psychologist in doctoral training). An interpretation service was offered for those who needed, or preferred, to speak in their native language. All participants opted to speak in English. Interviews were audio recorded and ranged between 25–60 min in duration.

#### Reflexivity

A bracketing interview was conducted prior to participant interviews, which aimed to identify prior assumptions, biases, and expectations of the researcher in engaging with the research subject to minimize the impact of personal prejudices on the procedure and results. Each participant interview was followed by a reflexive discussion between the researchers to reflect on the process of interviewing, consider participant well-being, explore any safeguarding concerns, and discuss any adjustments necessary to facilitate the telling of rich, elaborate narratives. All interviews were considered of adequate quality for inclusion.

### Ethical Considerations

Ethical approval was granted by Queen’s Square Ethics Committee. Participant well-being was prioritized at all stages of research in line with the British Psychological Society code of human research ethics (British Psychological Society, 2014). Participants were informed of the anonymity and confidentiality of their data, including the limits to this agreement related to the legal and safeguarding obligations regarding FGM.

### Data Analysis

Both researchers were experienced in qualitative data analysis. Analysis was informed by Smith et al.’s (2009) theory of IPA for larger samples and step-by-step guidance from Larkin and Thompson (2012). Stage 1 entailed line-by-line free coding of each transcript, allowing for the most striking components of the transcript to be captured. Stage 2 involved a more systematic clustering of objects of concern (things that matter to the participant) and a more interpretative layer of meaning developed. Stage 3 organized these into emerging themes, and Stage 4 identified recurring themes across cases, which were categorized into superordinate and subordinate themes in an iterative process of review and adjustment to find the most meaningful organization of themes. Credibility checks were employed through supervision and consultation with an expert in IPA.

## Results

### Situating the Sample

Nine women who met the inclusion criteria agreed to take part in the study. Table 1 summarizes the demographic characteristics of the group.

**Table 1.** Participant Demographic Characteristics.

Age	Range = 26–57 years	*M* = 38.3 years
Country of origin	Somalia (*N* = 4) Sierra Leone (*N* = 4) Yemen (*N* = 1)	
Length of time living in the United Kingdom	Range = 11–20 years	*M* = 16.8 years
Employment status	Employed professional (*N* = 9)	
Marital status	Married (*N* = 8) Divorced (*N* = 1)	
Length of time in relationship	Range = 2–20 years	*M* = 8.7 years
No. of children	Range 0–4	
Pregnant Y/N	Pregnant (*N* = 3) Not pregnant (*N* = 6)	
Type of FGM	Type I (*N* = 1) Type II (*N* = 2) Type III (*N* = 4) Unknown Type I or II (*N* = 2)	
Estimated age of FGM	Range = 2 weeks–17 years	*M* = 8 years

*Note*. FGM = female genital mutilation.

All participants had migrated from their countries of origin and had lived as working residents in the United Kingdom for a considerable length of time and all had good or excellent English language ability. All were either married or divorced. All but one were mothers or soon-to-be mothers; three women were pregnant at the time of interview. There was a range in FGM type reported and two women were unsure or there was a level of unclarity. Of the four women with Type III, all had undergone deinfibulation (opening) procedures in the United Kingdom. There was a wide range in estimated age at the time of FGM and also in whether or not the event was remembered. Each participant was randomly allocated a pseudonym that will be used throughout the article to preserve anonymity. Verbatim quotes will be used to illustrate themes.

### Themes

Three superordinate themes emerged through the data analysis alongside 12 subordinate themes displayed in Table 2. The first superordinate theme describes the conflict that participants experienced in deciding whom to blame for their FGM, while attempting to preserve their parents as good. The second superordinate theme represents the process of positioning oneself in relation to important people, creating a sense of being lucky or unlucky in comparison. The final superordinate theme captures women’s attempts to regain power from a previous state of powerlessness and to find strength from a place of vulnerability to put right in adulthood what was deemed to be wrong in childhood.

**Table 2.** Overview of Themes.

Superordinate themes	Subordinate themes
The “who to blame?” conflict: Preserving goodness in parents	The benevolent parents who failed me
	The anonymous cutters
	The struggle with self-blame
	The power of culture
Better or worse? Positioning the self in relation to others	Indebted to the “good husband”
	Comparison with other women
	Belonging or exclusion
	My place in the family
Regaining power: Righting the wrongs	The unprotected become the protectors
	From unknowing to knowing
	Emancipation from taboo
	Renewing purpose in pain

### The “Who to Blame?” Conflict: Preserving Goodness in Parents

All participants presented in their narratives an apparent need to locate blame for their FGM. FGM was strongly condemned and perceived as a deliberate, relational act with an originator, a decision-maker, and colluding bystanders. At the forefront of blame were the parents as the ones responsible for making decisions for their children. However, the situation becomes complex and blame shifts between those who are known (parents, grandparents, and the self) and those who are unknown (“cutters,” wider culture) who are less readily absolved.

#### The benevolent parents who failed me

Some participants expressed anger toward their parents for organizing their FGM or for failing to protect or prepare them. Particularly guilty were mothers; fathers were less present in narratives in comparison, although at times they were blamed for not exerting their authority and protecting them from the mother or grandmother:

My Dad always lets my Mum have it her way but yeah . . . um . . . that one wasn’t a good one, I think my Dad should have stopped her. (Aisha, 471)

Grandmothers were also absolved of their involvement, which was influenced by most of them being deceased and thereby were forgiven. Attitudes changed over time with maturity and the influence of critical life events, such as becoming a parent oneself, asking parents questions, or receiving an apology or expression of regret:

That changed through the years, yeah that has changed because um . . . I have realised that my mum felt so bad of what I went through personally . . . because when I was a teenager she will call—she will see me go through a painful life, like having pain all the time and she regret a lot . . . she regret because she said I didn’t know better . . . I didn’t know better . . . otherwise I would not have done that. (Maymun, 381)

The nature of the intentions behind the parental decision was deemed important. Relief was found in the belief that parents had good intentions, rather than acting maliciously. Initial confusion, anger, and feelings of betrayal often turned to acceptance and forgiveness, with greater sympathy for their parents’ fallibility, lack of education, and societal pressure.

I never thought that my Mum actually done something bad . . . but when she . . . apologised for it and she was talking about it . . . after the phone call I had to sit down and think about it, and I was like . . . wait, was it bad? Like what’s wrong with me now? I felt like there was something wrong with me [laughs]. (Aisha, 364)

Aisha alludes to a possible function of preserving the parents as good to simultaneously preserve the self as good, particularly in the context of strong identification and close relationships with mothers.

#### The anonymous cutters

The “cutters” who performed the FGM were frequently mentioned but were almost always unknown, sometimes unseen due to the girls being blindfolded. They were dehumanized and described as brutal, faceless aggressors. Their qualifications were important; either they were unqualified and therefore illegitimate, dangerous, and irresponsible, or they were educated professionals such as nurses or midwives and should have known better than to hurt children for whom they ought to be caring:

The nurse itself—herself, sorry . . . um . . . you should be educated. Why are you doing this? Why are you getting money to hurt people instead of making sure people are safe? Those kind of questions will go in my mind, thinking . . . and then I was getting angry as I talk and saying like . . . I don’t know . . . yeah. (Maymun, 364)

Cutters were not absolved of blame and it may have been preferable to participants to direct anger and aggression here rather than toward the parents and wider family.

#### The struggle with self-blame

Most women described a wholly nonconsensual FGM experience, particularly those at a younger age. Some, however, recalled insisting that their parents let them have FGM, or going there willingly. This was not an informed choice but a desire to be like everybody else and join in an exciting ceremony such as in the “Bondo” society of Sierra Leone. There was a tendency among participants to apportion blame to themselves for their FGM directly or for their inability to cope with the consequences or the impact on others. There seemed to be a tension between taking some blame away from parents but not wanting to take all of the blame due to the negative impact to the self. Blame could be directed outward instead:

I don’t know. I—I know she didn’t want it. So I can’t actually blame her a lot. Although as I—as I said it was like a social norm. Kind of I wanted it as well, so I won’t actually blame her. First of all, she doesn’t want it, and it’s a social thing that you want to get belong to a certain group, so I can’t blame her. I can’t blame myself, it’s just . . . one of those things that . . . if education was there, if you were educated . . . (Marai, 282)

#### The power of culture

Women described powerful social forces to which their parents were subjected. Religion was protected from blame but wider “culture” and ancestral “tradition” were held strongly accountable for parental impotence regarding FGM. Parents were perceived as doing the same as everyone else, trying to do right by their families in the context of FGM being the social norm:

A lot of these people it’s not their fault, they’ve got nothing to do with it, but it’s what tradition and society has succumbed them to do, and that’s what happened. (Favour, 671)

There was an inevitability and inescapability to FGM in this powerful cultural context, with individual resistance feeling futile. Intergenerational and community pressure were thought to be strong, and there was a desire in some to locate the ultimate originator of the practice. There was also recognition of how culture has changed over the course of their lifetimes, particularly with regard to education about the health consequences of FGM, evoking a hopefulness for change in cultural norms.

### Better or Worse? Positioning the Self in Relation to Others

Women continually positioned themselves in relation to important people in their lives. This seemed to reflect a confronting of the question, “Am I lucky for surviving my ordeal or unlucky for being afflicted in the first place?” Negotiating this dilemma seemed to have implications for one’s sense of self-worth and belonging in social systems.

#### Indebted to the “good husband.”

The husbands of participants were described in strongly favorable terms: as supportive, understanding, and loving. These qualities were contrasted with the way women described themselves, as though they were failing in their role as wife. Stories of sexual inadequacy were commonplace, with women exclusively locating the problems within themselves by way of pain and discomfort, coldness and avoidance, lack of desire, and absence of pleasure and enjoyment. Transparent communication about FGM being the reason for this situation protected them against husbands feeling rejected or unloved and maintaining marital harmony. Husbands were credited with being patient, dedicating extra effort to enable her to achieve pleasure and remaining faithful rather than leaving her for an “uncut” woman. Women said they felt grateful and lucky to have such good husbands and told stories of other men whom they said could be pressurizing, forceful, and unfaithful.

I was lucky, I got someone who was very understanding so . . . it all comes down to him that he is able to cope with it. If that is actually affecting my relationship with my husband, if he left me because of that, then I’ll have gone back and said Mum, see . . . (Marai, 494)

This quote suggests that this husband is credited not only with good qualities, but also with protecting his wife against a more severe interpersonal impact and confrontation with the mother.

#### Comparison with other women

Women evaluated the impact of FGM on their lives by way of comparison with other women. Frequently told were horror stories of death, tragedy, and more severe complications than their own. Relief was found in feeling “lucky” in comparison with other women and having escaped something much worse:

I must say I have been lucky in the sense that I didn’t have most of the complications that women do have and suffer with. (Fatima, 381)

This seemed to be a way to bolster self-worth. Women made individual comparisons, particularly with sisters and friends, and compared their country with the practices of other countries:

The practice in Sierra Leone is not that bad as the other like some other countries where they cut and they sew and all of that. (Idil, 650)

Different FGM types were also evaluated by comparisons. This woman described feeling her Type III was worse than her Type I counterparts:

Actually . . . I think three and two . . . one is better than that. One is actually they just take the clitoris, but it won’t cause you any harm or . . . difficulty of giving birth or also you see more infection because of they take out everything. It’s very hard. (Najma, 104)

Najma seems to say that she, unlike others, has had “everything” taken from her. Feeling worse off or unlucky in comparison with others seemed to evoke a painful sense of inferiority or shame. Women who felt unlucky also held a sense of being damaged or abnormal. Migrating to the United Kingdom was thought to impact on comparison tendencies as the environment changed to one where FGM is no longer considered the norm:

And um, it’s very painful really I mean . . . I feel like . . . [sigh] I’m not normal like other ladies . . . that’s why I ask . . . if they can do anything about it, they can do they can repair the . . . this damage, I know it’s very . . . I’m old [laugh] but I feel like I want to live like normal people. I want to live, I want to feel. I want to feel like other people feelings. (Adama, 116)

#### Belonging or exclusion

Women provided insight into a need to belong in one’s community: to feel sameness, connected, and secure in one’s in-group. Exclusion was considered a worse outcome than physical pain, which was a frequently reported reason for the perpetuation of FGM within societies where it is considered the norm and “uncut” girls are ostracized and shamed. This situation reversed for the sample after migration to a nonpracticing country. FGM was treated by some as a shameful secret, hidden from view, and there was a fear of rejection, judgment, or attack for being different. This fear related most strongly to friendship groups and potential partners:

I felt different in terms of . . . getting married, I was so scared to get married, thinking . . . what is gunna happen when I get married? What the person gunna say? They gunna say what happened to you and you know . . . I was so scared . . . for me to even think about to get married or have uh . . . sex with someone, always thinking there’s something wrong down there and they will find out and they will tell me like things . . . (Maymun, 199)

Another participant believed that her divorces were directly linked to her sexual failings caused by FGM, and that she subsequently suffered with an intense fear and anticipation of repeated abandonment in relationships.

#### My place in the family

Women’s narratives emphasized the importance of responsibilities associated with their position in their family structure and hierarchy. Although inevitably culturally and individually varied, women presented a set of assumptions about family roles. For instance, older sisters took care of younger sisters, boys protected girls, and fathers were largely absent although they held authoritative positions. Children belonged to their parents. It was not acceptable to act against one’s parents. This, of course, also applied to one’s grandparents, raising the question of who runs families, parents or grandparents? These implicitly understood social rules not only seemed to help make sense of relational dynamics, but also raised confusion when expected roles were not fulfilled—for example, when protectors were not protecting, or when authority figures were impotent. Idil considers the impact of grandparents’ wishes on her parents’ decision to allow FGM and how positions changed once the grandparents passed away:

They sort of did it as a form of loyalty to their parents, to honour the last thing that they wanted from their grandkids before they die. So they sort of like they did that so I don’t know if because that burden wasn’t on them anymore, to like please anybody or their parents, so they didn’t just go there anymore. (Idil, 133)

### Regaining Power: Righting the Wrongs

The women strongly condemned FGM, regarding it as a moral issue in which they had been wronged; a wrong that needed to be put right somehow. Some gave lengthy, descriptive accounts of their FGM experience, vividly portraying their powerlessness in the face of serious danger and disablement. Women depicted themselves as survivors, having endured unconscionable pain without medical aid, reflecting a view of themselves as strong, while mourning for their lost childhood innocence. Acts of resistance took many forms and there was a striking focus on creating something good from suffering, rather than seeking revenge or perpetuating wrongdoing.

#### The unprotected become the protectors

Women described a situation in which they were unprotected from the dangers of FGM in childhood. Some talked of entering into their “initiation ceremony” blind, blissfully ignorant, and how this was felt to be exploitation by a system of knowing adults. The people whom they expected to protect them from harm—parents, grandparents, nurses, community elders, and friends—failed to do so. Women described how, as they grew up, they assumed the role of protector themselves. They all exhibited a fiercely protective stance toward their own children (or future children) and voiced a strong desire to “save” younger members of their family and the younger generation of girls more widely. Taking an authoritative position, engaging in activism and campaigning, and shielding their own daughters from FGM, they became the wished-for parents and protectors, and disrupted the intergenerational transmission of powerlessness:

Um . . . I’m glad I know now, because in the life if I’ve got child, I will never put through my child ever or like . . . somehow it was a sign for me to having this . . . so at least . . . if any my family or anyone else, I could literally stop . . . I could at least save someone . . . you know what I mean? (Halima, 484)

#### From unknowing to knowing

Some women had vivid memories of their FGM experience and described attempts to suppress and forget. Others were too young to remember and learning about their FGM in adulthood was a significant event, often difficult to process. There was a conflict of knowing; on one hand, it was helpful to understand but, on the other hand, considered a painful burden. This woman believed that continued avoidance of knowing would result in a continued pattern of abandonment in her romantic relationships:

I wish I didn’t know. But in . . . somehow I wish I didn’t know and somehow I . . . it’s better to know . . . if I got married to this person or we have any kind of relation . . . he would . . . he will leave me . . . and I will go back to the same circle asking why and why. (Adama, 266)

Avoidant defenses were commonly described to prevent knowing (avoidance of one’s own genitals, and suppression of thinking, remembering, or talking about it). At the same time, many women showed a clear pursuit of knowledge about FGM. There was a desire to be educated about the association between FGM and symptomology. Once educated, women put great faith in the power of education to stop the practice of FGM and help others:

I think we just need more education, just to educate people. It will change. ’Cause once you empower the young ones . . . (Marai, 180)

There was a belief that if only their parents had been educated about the harms of FGM they could have been spared. This idea of knowledge as power could also be conceptualized as an act of resistance against the secrecy and blinding experienced as a child.

#### Emancipation from taboo

The taboo surrounding FGM had a strong presence in narratives. Taboo was described as an oppressing force embedded in culture and strictly, even violently, enforced through manipulation, coercion, and “*brainwashing*” (Fatima, 197). Some women described how the societal silencing of FGM discourse could cause disconnection in relationships with husbands, siblings, parents, and friends, and breaking the silence was both frightening and freeing. For some, speaking out about FGM risked real physical danger, and migration allowed increased freedom. Numerous modes of expression were found in support groups, psychotherapy, public speaking, and sending family members news stories or pieces of research. Talking about FGM could be painful but was generally considered helpful:

It helps a lot, it ease that tension you know it ease that pain, it ease that burden that you feel now, because before people don’t talk about it, you feel shy to even talk about it, to say I’ve been through it, you know, even though . . . we know . . . that it’s only now that FGM came in the open that people feel happy to talk about it. Before because even when you go through that, in there, it’s a taboo. They say to you, you do not ever talk about this when you come out. So you never ever talk about it. You’re a child. Obviously if they say that to you what would you do? You keep quiet, you know. So it’s only now people feel free, people are happy to talk about their experience, share their experience and things like that to be honest, which I’m glad about to be honest. I’m really, really glad about that, ’cause you know you just feel all bottled up, you’ve got that heavy thing in your heart, you can’t talk about it, you can’t . . . but now you can talk about it anywhere, I don’t care where I talk about it. (Favour, 593)

This theme was mirrored in the research interview process; at follow-up, many participants described finding the interview therapeutic, saying it was not usual for them to have a space to freely express personal feelings about taboo topics such as FGM and sexuality, and that they felt strongly about being able to give something to help others through telling their stories.

#### Renewing purpose in pain

A fundamental difference between purposeful and purposeless pain emerged in women’s accounts. FGM for some incurred lifelong physical and emotional pain, and was considered by many to be devoid of purpose; the justifications available were deemed unacceptable. In contrast, women found great purpose in the pains of childbirth and motherhood, which represented womanhood and naturalness that were deemed very important:

Yeah because I really want everything natural, I don’t want the gas and air or anything . . . like I wanna . . . experience—this sounds a bit weird but, the pain I don’t know [laughs], like I wanna have my child naturally so that I can talk to her about like . . . I don’t want . . . ’cause I’ve seen it cause . . . and in some . . . cases I’ve seen some female when . . . they’ve been asked to push and stuff . . . like they don’t know what’s going on, they have to be told like, the midwives that are looking at them telling them OK you’re in this much pain, I wanna know what’s going on, don’t want anybody to tell me, I think maybe I’m a control freak a bit init—I wanna be in control of my feelings [laughs], I wanna experience the pain like that . . . um so yeah . . . no . . . I’m gunna go for it. (Aisha, 634)

Childbirth stories like Aisha’s seem to reflect a mastery of a previous state of powerlessness. Similarly, deinfibulation was perceived as a purposeful choice, painful at first but representing openness and liberation. Motherhood was depicted as an opportunity to be a creator, an educator, and to be responsible for one’s child, and a new beginning with the chance to do things differently from one’s own parents.

## Discussion

This study used IPA methodology to explore the relational dynamics of women who experienced FGM in childhood and migrated to the United Kingdom. Three superordinate themes emerged: capturing a conflicted interpersonal blaming process, a positioning of the self in relation to others, and a regaining of power from a previous state of powerlessness. The findings will be discussed in light of existing theoretical and empirical literature. Limitations of the study and real-world implications for policy and practice will be considered.

### Discussion of the Findings

Women were greatly concerned with attributing blame for their FGM but faced a tension between the need to blame and the need to preserve one’s parents as good. Parents were largely absolved of blame through justifying and sympathizing with their impotence; however, cutters and wider culture remained guilty and some self-blame was reported. This is a similar finding to the blaming process seen in Parikh et al. (2018), whereby women found forgiveness for their mothers and placed blame on wider culture, which was also interpreted as a way of salvaging the disrupted mother–daughter relationship.

Blame is an inherently social process; it attributes badness to another person and makes judgments on their intentions and moral character (Nadler, 2012). It makes sense for women to emphasize the good rather than malicious intentions or negligence of their parents as it is in their own interest to preserve these important relationships. The tendency of children to blame themselves for traumatic events has been frequently reported in studies of children sexually abused by a primary caregiver (e.g., Quas et al., 2003). Self-blame may serve an adaptive function in the face of inescapable abuse to create a sense of control in the face of powerlessness, to maintain some attachment to a caregiver who is depended upon for survival, and to preserve some remnants of trust in others (Gleiser, 2003) and belief in a just world (Lerner, 1980). In the long term, however, such defenses and distortions can leave individuals vulnerable to psychopathological outcomes and unconscious reenacting of patterns of victimization in adulthood (Mokma et al., 2016).

Whereas FGM is classified as child maltreatment by international definitions (United Nations Children’s Fund, 2016), such concepts are influenced by historical and cultural ideas of well-being, child development, and parenting, and there is a contentious debate between ideas of cultural relativism and absolutism that speaks to this tension (Wade, 2012). This creates a complex situation when women, such as the participants in this study, do not perceive their parents as abusers, but rather as loving parents, conforming to a particular time and culture. Women may not identify as “victims” of abuse but have migrated to a country that labels them as such; “mutilation” carries a stronger demand for blame than the more neutral “circumcision.” Such labeling, therefore, may add strain to an already conflicted mother–daughter relationship.

Attributing the majority of blame to “culture” or “tradition” appeared to ease this tension for participants. If blame poisons relationships, it may be safer to direct it toward faceless objects or systems. Nonetheless, blaming “culture” is not without interpersonal consequence. Shunning or denigrating an aspect of one’s culture may pose a threat to one’s identity and sense of belonging, particularly in the context of settlement into a Western culture, and increase the need to redress self-worth by comparison with others. Participants continually positioned themselves as better or worse in relation to others. They reflected upon their need for belonging and connectedness, and the consequential pain of exclusion, rejection, inferiority, and abandonment when this was not met. The psychological need to belong is ubiquitous and part of the essence of humanity (e.g., Maslow, 1954). It depicts a feeling of being “at home” (Antonsich, 2010) and is therefore challenged when an individual migrates to another country and has to reassess and renegotiate one’s identity and membership with different groups (Anthias, 2016). Participants had a strong sense of family duty and hierarchy, which appeared to be important to preserve despite geographical distance, most likely to maintain a sense of belonging in one’s family and original community concurrently with one’s new environment.

Interpersonal relationships also contributed to women’s sense of good fortune or “luck.” On one hand, women reported feeling lucky compared with others. Feeling lucky may be a characteristic of survivorhood, serving to defend against victimhood and its associated inadequacy and vulnerability. Whereas victimhood evokes powerlessness, survivorhood can evoke a prosocial sense of duty to help others less fortunate, as seen in participants’ engagement in activism and helping younger members of their family.

On the other hand, women could feel unlucky, different, or inferior. Fear of rejection, particularly by men, was offset by gratitude and indebtedness toward their husbands, who supported and stayed with them, despite a range of perceived sexual inadequacies. Here, women described being lucky in their unluckiness. This relational imbalance may perpetuate a patriarchal power dynamic in which women are emotionally, and/or otherwise, dependent on husbands due to a perception of being “damaged.” A psychoanalytic view put forth by Kulish (1991) posits that the clitoris is feared by men due to fantasies of its phallic power and of uncontrollable female sexual impulses making uncut women impossible to satiate. The patriarchal solution is to deny its existence in discourse, making female sexuality shameful, immoral, or taboo, or to enforce its suppression by literal excision. This reduces the need to satisfy the woman, thereby sparing men of narcissistic mortification (Lax, 2000). In this study, such mortification appears to be located instead in the women. Abusharaf (2001) challenged the widespread perception of women as victims of male dominance, stating that women who have been cut sometimes exert an enhanced sense of power and authority in marital relationships. This view was not substantiated in the present study results. Future research with couples could further elucidate the dynamics of shame and power imbalances within the functioning of marital relationships.

Women strove to regain power in adulthood, and relational dynamics changed as they grew up and took on different roles in their family structure. Far from presenting as victims, women not only survived experiences of extreme disempowerment, but also they later thrived in coping with pain and adversity, helping others, speaking out despite powerful barriers of silencing and secrecy, and campaigning and advocating social change, thereby challenging a reductionist narrative of adversity leading to only psychopathological outcomes. That is not to deny the potential traumatization in this population but to acknowledge the ways in which adversity can also engender significant strength. Some women gained a sense of mastery over powerlessness through the childbirth event, most likely due to its similarities with the FGM experience—for example, repeat cuts, extreme pain, feeling helpless at the mercy of professionals, and a period of healing and recovery—a way of repairing wounds in the present that cannot be changed in the past and finding meaning in otherwise “purposeless” suffering.

Women reported disrupting the intergenerational transmission of FGM by protecting their daughters and the younger generation at large. They resolved to do things differently from their ancestors and took upon themselves the responsibility to do for their children what they wished their own parents could have done for them: to protect, educate, and exert authority. Indeed, Boyle and Svec (2019) found that women with autonomous or joint household decision-making powers were more likely to abandon FGM for their daughters.

Women may have felt able to regain power because they were able to face the pain of their experiences: to bear the burden of knowing, rather than sustain avoidance or dissociation. Migrating to the United Kingdom may have made it more difficult to avoid the issue. Joseph (1996) suggested that, in order for women to continue to cut their daughters, they must have to deny the memory of their own suffering and rage. Mothers may not even perceive their own pain in childhood and children are cut under the guise of being protected. Indeed, in Schultz and Lien (2014), mothers perceived protection as not letting children know what will happen to them. Yet the women in this study described feeling unprotected and let down by this failure of preparation. Afifi and von Bothmer (2007) found that women who do not use dissociative mechanisms were more likely to condemn FGM and less likely to continue it. This seems to be the case for the current sample who attributed power to education and knowing. Arguably, only after pain is acknowledged can anger and mourning, strong motivators for growth and social change, begin.

### Implications

This study adds depth to understanding the relational dynamics of women who have experienced FGM. It took a holistic, rather than pathologizing, orientation and focused on subjectivity of experience. This contributes to an ethos of understanding before explaining complex phenomena (Leonard, 2000) that can be applied broadly in developing clinical practice and policy around working with FGM populations.

This study contends that psychological well-being is socially situated and community-based interventions may need to be considered for women who are afflicted by social isolation, feeling different or damaged, feeling silenced and unable to access help, or experiencing strain in marital relationships.

For those who seek psychotherapeutic intervention, the current findings may provide clinicians with a sense of the complexity of issues associated with FGM and help them avoid taking a stereotyped view of women as victims, which can perpetuate systemic disempowerment. Studies exploring practitioners’ attitudes toward working with women with FGM report a significant gap in knowledge, understanding, and confidence in this area (Elliott et al., 2016; Jackson, 2017). One helpful psychotherapeutic stance would be to allow women to access and process their pain at their own pace and choice, and support them through the potential pain of knowing. Timing and readiness will be important factors for the suitability of different interventions such as individual, couples, and group therapy. A key point to assess and offer psychotherapeutic support is in gynecological and maternity clinics where FGM may be first identified.

Women identified that the typology of FGM (Type I to IV) could contribute to their tendency toward “better or worse” comparisons, and clinics ought to consider the support offered around diagnostic labels and recognize that medical terms are not neutral. Future research could further investigate women’s experiences of typological labeling, with reference to the broader issue of “mutilation” discourse and its personal and societal impact.

The findings have additional implications for understanding why some women choose, and feel able, to successfully disrupt the intergenerational transmission of FGM. Women identified a need for safety and agency to express their views and act more freely. Many had been supported to make meaningful contact with their pain rather than dissociating, which enabled them to seek knowledge and educate others. This highlights the importance of survivor-led training and education programs. Further research may elucidate the relationship between dissociation and discontinuing FGM, explore how migration and education impact on attitudes, dissect the reasons why some women cease to continue the practice, and help others to overcome powerful barriers to discontinuation while maintaining an important sense of cultural and familial belonging.

### Limitations and Future Directions

The findings of the current study must be considered in conjunction with a careful examination of the sample characteristics that limit generalizability. All participants were good English speakers, well-educated, and long-settled migrants working in the United Kingdom. Invitations for non-English speakers to participate with interpreters were not taken up, so this group was not represented. There will inevitably be stories that are silenced by fear of the repercussions. In addition, participants were recruited through an NHS clinic and a charity group that campaigns against FGM, and this is likely to produce a bias in terms of the stories presented. It is quite possible that those with positive or neutral attitudes toward FGM would describe different relational dynamics. The “pro-FGM” group is more difficult to access in the United Kingdom due to the legal implications of professing to have practiced or intending to practice FGM on one’s children and fear of judgment from professionals. This group is therefore underrepresented in FGM research, including in the current study.

Data on refusal and drop-out rate were not systematically studied but would be helpful in future research to further understand sample biases. The more “disempowered” and “traumatized” groups identified by Vloeberghs et al. (2012) are predicted to be more likely to avoid and distrust platforms available for speaking about their experiences. Different recruitment sites that encounter more marginalized women could be sensitively approached and safe, confidential ways of engaging in research advertised in potential participants’ native languages with trusted peers or professionals. In essence, future research must seek to understand the barriers for underrepresented communities not only because a plurality of voices can be heard in research and inform practice, but also because they are likely to be some of the same barriers that prevent some women from accessing health and social care services.

## Conclusion

The current study provides insight into the relational dynamics of adult women who have experienced FGM in childhood and since migrated to the United Kingdom. An IPA of women’s narratives identified a conflicted blaming process in which the need to blame someone acted in tension with the need to preserve one’s parents as good. Women also engaged in a process of comparison with others as better or worse off, which helped them negotiate a sense of identity and belonging, particularly salient in the context of migration. Finally, the women regained powerful roles in their lives from a previous place of powerlessness and held a strong sense of duty to protect the next generation of girls. The findings hold valuable implications for clinical practice and policy. Findings should be interpreted in the context of important sampling biases and future research is recommended to investigate barriers for more marginalized or silenced women to share their stories on FGM as a part of their personal and interpersonal lives.
